# Three-dimensional Layered Water-Soluble Cellulose Acetate/Polyacrylamide Composites with Ultrahigh Ductility and Stretchability

**DOI:** 10.1038/s41598-017-13374-4

**Published:** 2017-10-16

**Authors:** Jinhui Pang, Miao Wu, Xin Liu, Bo Wang, Jun Yang, Feng Xu, Mingguo Ma, Xueming Zhang

**Affiliations:** 1 Beijing Key Laboratory of Lignocellulosic Chemistry, Beijing Forestry University, Beijing, 100083 PR China; 2 College of Marine Science and Biological Engineering, Qingdao University of Science & Technology, Qingdao, 266042 PR China; 3 National Engineering Laboratory for Pulp and Paper, China National Pulp and Paper Research Institute, Beijing, 100102 PR China

## Abstract

Water-soluble cellulose acetate (WSCA), one of the most important cellulose derivatives, possesses biocompatibility, biodegradability and broad chemical modifying capacities. In this work, highly polymerized WSCA was firstly synthesized and used as cross-linker to fabricate highly ductile, tough and resilient WSCA/polyacrylamide (PAM) composite hydrogels. The results showed that the WSCA/PAM nanocomposite hydrogels exhibited extraordinary toughness and ductility with a tensile strength of 297 kPa and elongation at break of about 4020%. The enhancement of mechanical properties and stretchability were due to the synergistic effect from the hydrogen bonding and physical entanglement between the composite matrixes. Under stretching conditions, hydrogen bonds and the dense entanglement between WSCA chains and PAM could dynamically break and rearrange to dissipate energy. At the same time, the filaments of PAM embedded in layered WSCA matrix became unfolded or fractured to dissipate energy and maintained the conformation of hydrogels. It was envisioned that the introduction of WSCA into polymeric matrix would generate a facile method to fabricate multiple layered hybrid hydrogel network and significantly widen the WSCA applications in the preparation of high performance supramolecular systems.

## Introduction

Hydrogels, are highly customizable as three-dimensional networks of hydrophilic polymer chains, which can retain a significant amount of water^1^. Based on their flexible synthesis techniques and special soft-wet physical characteristics, hydrogels can be a good candidate for various applications ranging from biomedicine to daily chemicals, e.g., tissue engineering, drug delivery systems, sensing technology, and superabsorbent in diapers^2–5^. However, conventional synthetic hydrogels exhibit relatively low mechanical strength, which can’t sustain large deformations under tension or compression. Herein, their application scopes are severely limited due to their poor mechanical properties. Therefore, there are increasing demands for reinforced-hydrogels with enhanced mechanical properties^6^. In recent years, to overcome this limitation and extend their practical applications, numerous strategies have been developed to enhance the mechanical properties, such as double network (DN) hydrogels^7^, topological (TP) gels and nanocomposite (NC) hydrogels^8,9^. DN hydrogels are composed of a stiff and brittle first-network and a soft and ductile second-network with the enhanced mechanical properties. While, DN hydrogels utilize the sacrificial rigid network to dissipate energy through its fracture upon loading, showing a large hysteresis and residual strain in the tensile loading-unloading curves^10,11^. NC hydrogels use nanoparticles such as clay^12^, silica^13^, graphene^14,15^, and carbon nanotubes^16^ as cross-linkers. These nanoparticles as reinforcement elements are homogeneously dispersed in the polymer matrix with chemical bonding or physical adsorption to polymer chains, which have been demonstrated effectiveness in enhancing mechanical properties^7^.

Recently, tough and recoverable hydrogels have been synthesized based on polysaccharides as reinforcements, including alginate^14^, cellulose^2^, and agarose^17^. Among these polysaccharides, cellulose is the most abundant, biodegradable and biocompatible polymer on the earth^18–20^. Due to its unique physicochemical and mechanical properties, cellulose nanocrystal (CNC) as the hydrogel reinforcement has been widely reported^12^. While, strict size control of this nanoparticle and uniform dispersion in water were the crucial steps before utilization. Furthermore, the dispersion of nanoparticles in the aqueous system belongs to two-phase (solid-liquid) system and the contact areas were limited by the gross geometry of those solid nanoparticles, which, in turn, resulted in poor adhesion with the composite. Fortunately, water-soluble cellulose (WSC) can well solve these problems, as WSC can be completely dissolved in water, leading to form a homogeneous cellulose/polymer monomer system. During the gelation process, the dissolved WSC regenerated *in situ* to form a uniform network, in which the contact areas and interfacial adhesion were significantly increased. Generally, the degree of polymerization (DP) value of WSC was relatively low (DP = 200–300) as compared with that from virgin cellulose (DP = 800–900) due to severe degradation, which would substantially restrict the physical association between WSC network and polymer matrix, resulting in the reduction of the reinforcement of hybrid network^21^. Accordingly, synthesis of highly polymerized WSC would be favorable for the preparation of hybrid hydrogels with extraordinary mechanical properties.

In our previous work, water-soluble cellulose acetate (WSCA) with higher DP values (DP = 650–680) was synthesized via the side reaction in carboxylate ionic liquid^22^. Therefore, in the present work, fabrication of reinforced WSCA-polyacrylamide (PAM) composite hydrogels was investigated using the synthesized WSCA as the strengthening agent. The microstructure and mechanical properties of the hydrogels were characterized by means of SEM, swelling and strength tests. Moreover, the reinforcement mechanism was also elucidated. The results indicated that the introduction of WSCA polymer as the cross-linker could dramatically enhance the mechanical properties of the composite hydrogels.

## Results

**Figure 1:**
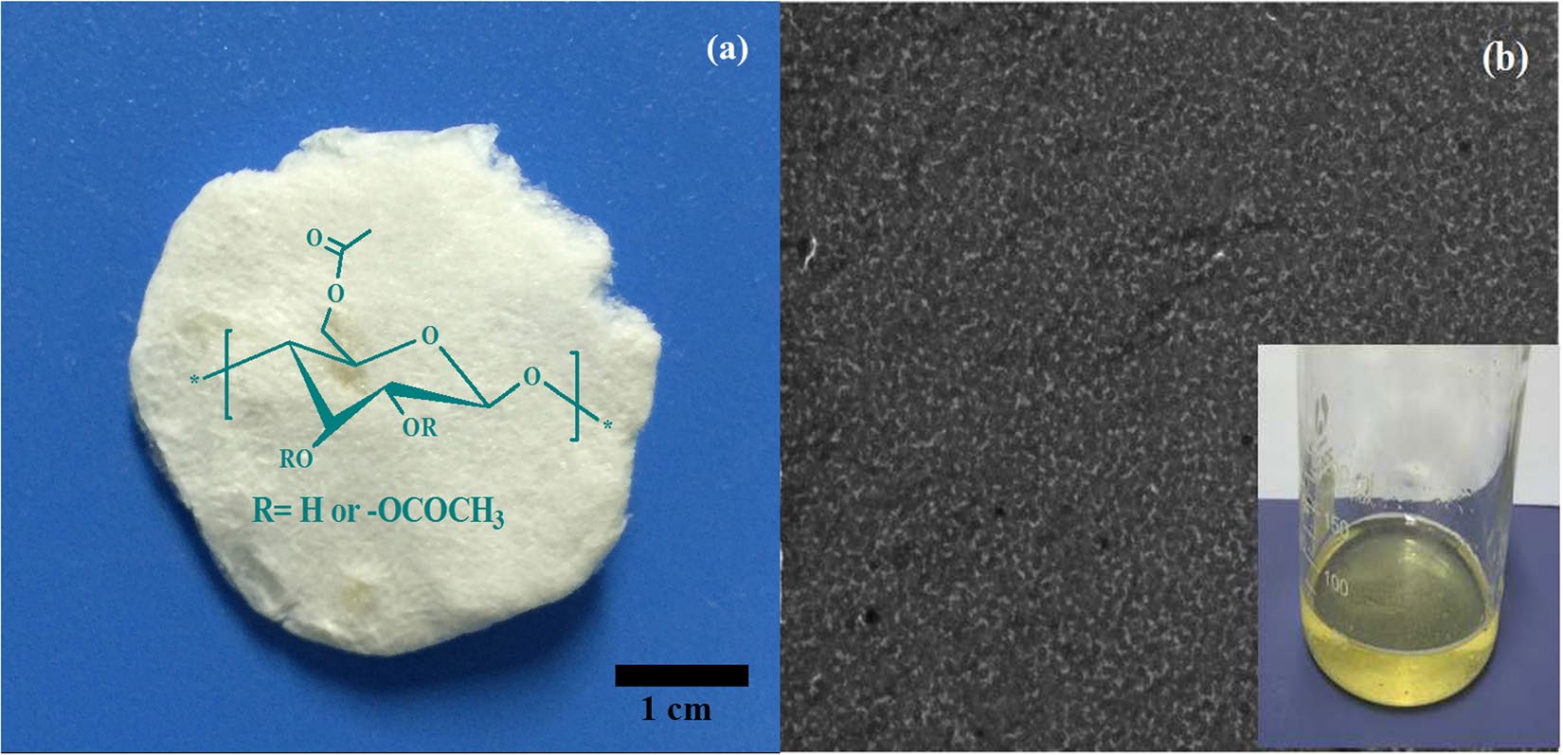
(**a**) Image of WSCA sample, (**b**) TEM of WSCA film.Inset is the solution of dissolved WSCA in water.

The WSCA with higher degree of polymerization values (DP = 650–680) was prepared from cotton linter using the methods as described previously^22^. Briefly, cellulose was dissolved in 1-ethyl-3-methylimidazolium acetate (EmimAc) and reacted with dichloroacetyl chloride (Cl_2_AcCl) in order to prepare cellulose dichloroacetate. However, under various conditions, a series of cellulose acetates with good water solubility were obtained. The synthesis mechanism based on NMR studies demonstrated that Cl_2_AcCl reacted with acetate anion of EmimAc, which led to create a mixed anhydride. Simultaneously, the cellulose was acetylated by the mixed anhydride, and the cellulose acetate was finally obtained instead of cellulose dichloroacetate. In this work, WSCA with DS = 0.63 was used as cross-linker and the image of freeze-dried WSCA is presented in Fig. 1a. As shown in the insert of Fig. 1b, 1% WSCA (weight, %)was completely dissolved in water and no fiber was observed, exhibiting superior water solubility. In addition, the homogeneous and smooth WSCA film was formed after evaporation of water as shown from transmission electron microscopy (TEM) in Fig. 1b. Obviously, the outstanding solubility of WSCA in water was favorable for the subsequent coordination with PAM matrix and made it as a potential superior reinforcement for the polymer-matrix composites.

**Figure 2:**
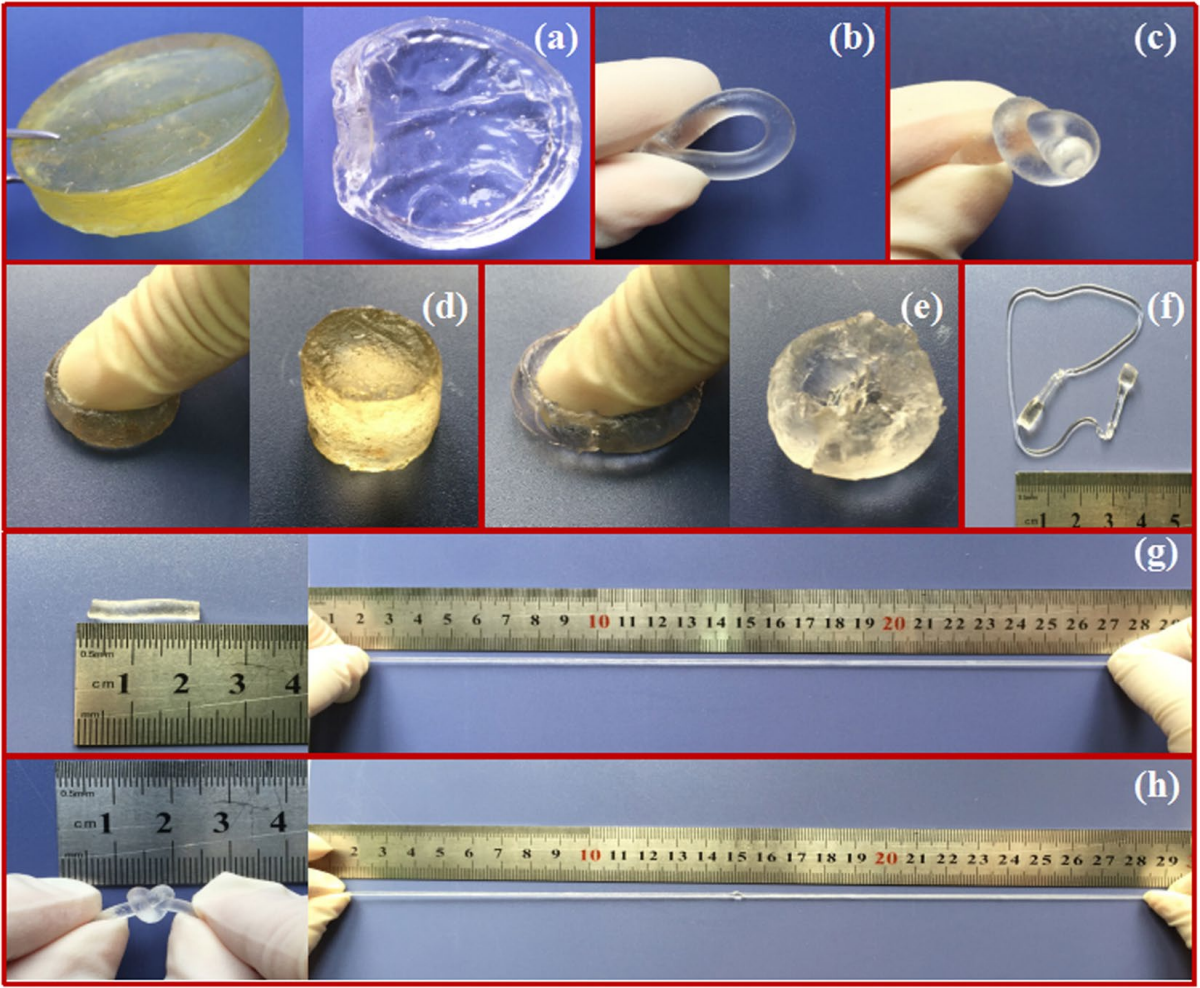
Photographs of hydrogels under different conditions: (**a**) Dried WSCA/PAM gel (left) and pure PAM gel (right); WSCA-PAM hydrogel being (**b**) Bent, (**c**) Knotted, (**d**) Compressed and recovered; Pure PAM hydrogel after being (**e**) Compressed and (**f**) Stretched; WSCA-PAM hydrogel being (**g**) Stretched over 20 times and (**h**) Stretched from a knotted state.

The processing scheme for the composite gels is shown as follows. Firstly, different proportions of WSCA (DS = 0.63) were dissolved in water, then the acrylamide (Am) monomer and ammonium persulfate (APS) were added into the aqueous solution followed by the consequent *in situ* free radical polymerization, resulting in the formation of the cellulose network-assisted composite hydrogels. In this strategy, acrylamide (Am) was the monomer, ammonium persulfate (APS) was the initiator, and WSCA was the cross-linker and strengthening agent. It was speculated that an abundance of hydrogen bonds could be formed between WSCA and PAM chains because a plenty of free hydroxyl groups in the surface of cellulose. Meanwhile, the water-soluble cellulose and PAM were miscible in water, which was favorable for the subsequent coordination, and thus leading to improve the mechanical properties. The enhancement for the toughness and stretchability of the hybrid gels by introducing WSCA are shown in Fig. 2. After drying under vacuum at 45 °C for 24 h, it was shown from Fig. 2a that the WSCA/PAM gel sample (left) still exhibited perfect cylindrical shape, while the pure PAM gel became curved and collapsed. Besides for the enhancement of dimensional stability, the introduction of WSCA could also dramatically improve the mechanical strength of the WSCA/PAM gels. The pure PAM hydrogels prepared without WSCA were readily broken by compressing and stretching because of its brittleness (Fig. 2e and f). However, the hybrid gels displayed a high performance in tensile strength and ductility as it could be bent (Fig. 2b), knotted (Fig. 2c) and compressed and then recovered quickly (Fig. 2d), which demonstrated that the tenacity property was dramatically enhanced. As shown in Fig. 2g, WSCA/PAM composite hydrogels remained unbroken as endured an extremely high strain, even in the knotted state (Fig. 2h) and it recovered its initial shape quickly after unloading. In addition, in was noted that the ductility of WSCA/PAM gels increased more than 20-folds compared with that of the unstretched state (Fig. 2g and 2h). Obviously, in these composite gels, the PAM belonged to the elastomers and acted as soft block, while the WSCA was considered as hard block due to its partially crystalline and amorphous structures. Herein, it was demonstrated that the mechanical properties and stability of WSCA/PAM composite hydrogels were dramatically enhanced due to the presence of hard block of WSCA.

**Figure 3:**
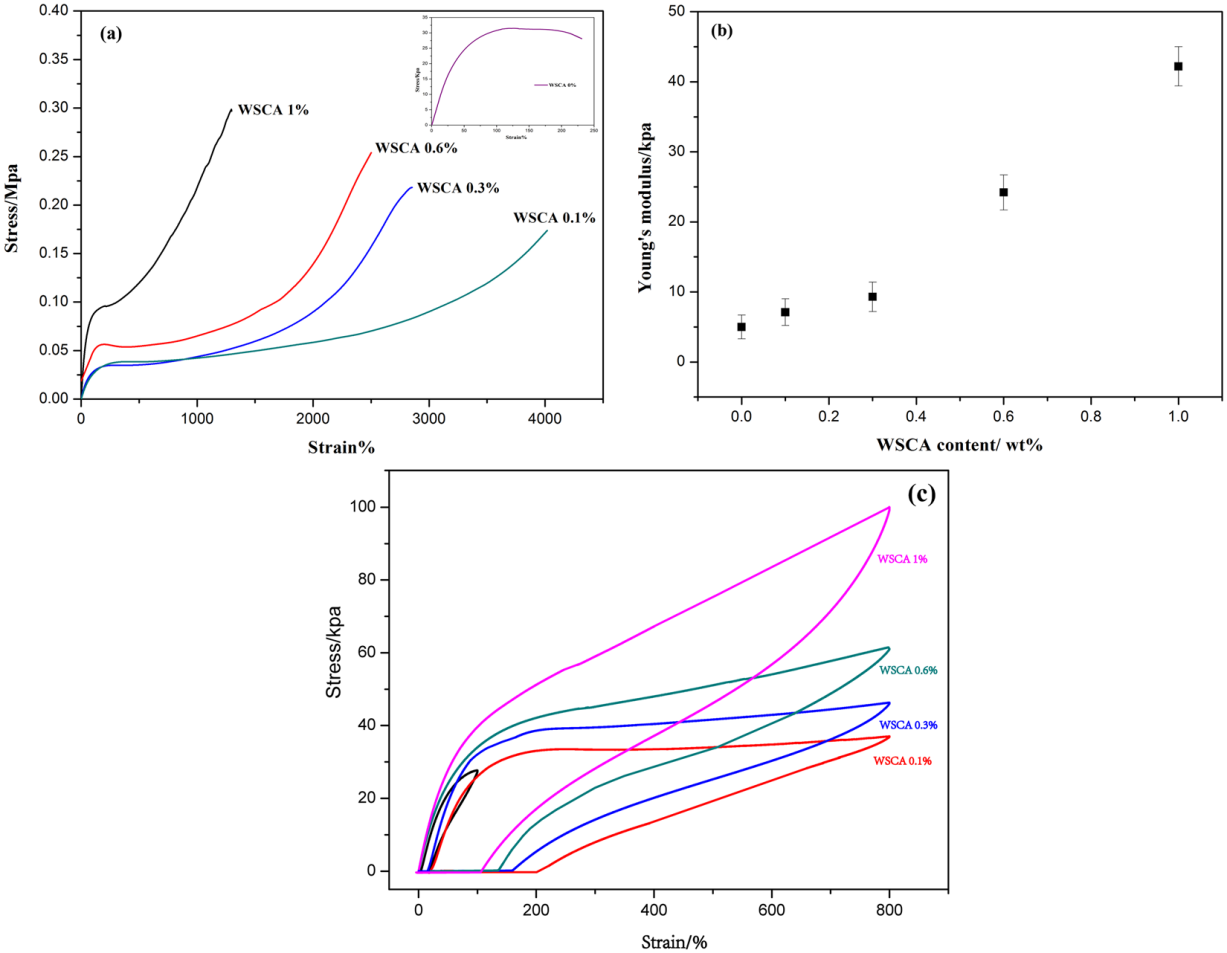
(**a**) Tensile stress-strain curves of hydrogels with different C_WSCA_ values, (**b**) Young’s modulus with different WSCA content, (**c**) Loading-unloading cycles with different WSCA contents at 800% strain.

Additionally, the monotonic and cyclic tensile measurements were also carried out to evaluate the mechanical properties of the composite gels. The typical monotonic stress-strain tests of the gels with different WSCA contents were performed and the stress-strain curves are displayed in Fig. 3a. As can be seen, the pure PAM gel (the insert of Fig. 3a) behaved rubber-like with much lower stress (27.6 kPa) and the elongation (231%) at break. While with the addition of WSCA, all the WSCA/PAM gels showed good deformation characteristics, capable of being stretched up to 17 times compared to the pure PAM gel (WSCA(0.1)/PAM sample with deformation 4020%). With increasing addition of WSCA, an increase in tensile strength and decrease in elongation could be observed, in which the tensile strength gradually increased from 174 kPa to 297 kPa, and the fracture strain decreased from 4020% to 1298%. In addition, it was also noted that the WSCA/PAM gels with less amounts of WSCA (samples WSAC 0.1% and 0.3%) still displayed typical elastomeric characteristic with no distinctive yield point, while it appeared in the samples of WSAC 0.6% and 1%, demonstrating the materials behaved more thermoplastic-like due to the substantial presence of hard block of WSCA. Moreover, it was observed in Fig. 3b that the Young’s Modulus increased almost linearly with the increase of hard block of WSCA content. According to some standard network theories such as neo-Hookean or Gaussian theories^23^, the significant improvements of mechanical properties and moduli were attributed to the formation of cross-linking net points between WSCA and PAM matrix.

To rationally guide the design of viscoelastic hydrogels with high toughness, it is a pivotal task to study the behavior of composite gels in dissipating stress energy^2^. Herein, cyclic stress-strain tests were performed to examine the recovery behaviors and stability of dissipative capability. The composite gels with different WSCA content were stretched to the strain of 800% and then recovered to zero load. As shown in Fig. 3c, with the increasing of WSCA, an increase in hysteresis loop and a decrease of unrecoverable strain could be observed, which demonstrated that superior energy dissipation ability could be achieved with the presence of larger amounts of WSCA. However, for the pure PAM gel, the hysteresis loop was much smaller and energy dissipation of the matrix was negligible.

**Figure 4:**
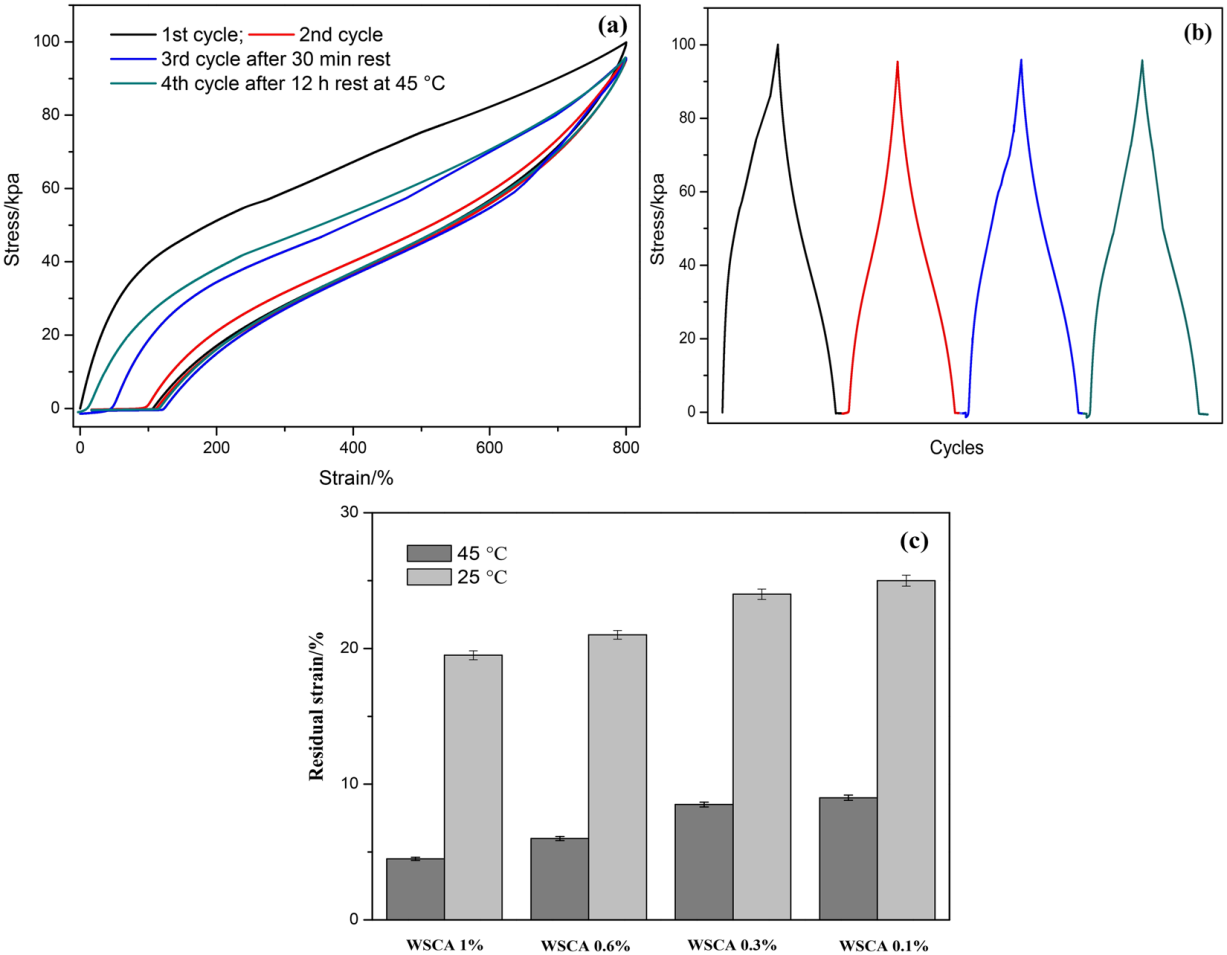
(**a**) Cyclic tensile stress-stain, (**b**) Stress-time curves of WSCA(1)-PAM hydrogel, (**c**) The residual strain of tensile tested samples with different WSCA content at different temperatures.

Moreover, the four continuous cyclic tensile measurements of WSCA (1)/PAM were also conducted to investigate the reversibility at a cross speed of 60 mm/min as shown in Fig. 4a. After the first cycle of loading-unloading, the next cycle was carried out immediately, then the third cycle was reloaded after 0.5 h rest, and the 4^th^ cycle was reloaded after 12 h rest at 45 °C. As shown in Fig. 4a, a prominent hysteresis was noted after the first loading-unloading cycle, indicating a high ability to dissipate energy during uniaxial stretching, which was presumably due to the rearrangement of the interaction between WSCA and PAM matrix and the deformation and dislocation of the WSCA network under stress. It was noteworthy that the hysteresis loop became negligible in the 2^nd^ cycle, in which residual strain reached about 110% after unloading. In order to investigate the recovery extent of the composite gel, the third cycle was conducted after 0.5 h rest at 25 °C. The results indicated that around 80% residual strain was partially recovered, leaving an unrecoverable residual strain of about 20%. Actually, this 20% unrecoverable residual strain was unrecoverable at room temperature after the 800% deformation. That was because the originally cross-linked segment was forced to move by the external force, when the external force was removed, the segment could not move anymore due to the ‘freezing-like’ deformation. However, the recovery of the segment could be partially resolved by raising the temperature^24^. Therefore, it was noted that the recovery was accelerated to eliminate the residual strain, which led to the nearly complete strength recovery at an elevated temperature at 45 °C (4^th^ cycle in Fig. 4a). This result further illustrated that the interactions between WSCA and PAM polymer chains only disassociated temporarily and there was no decomposition under stretching within the hybrid network as the matrix dimension could be easily recovered *via* polymer chains slippage at an elevated temperature^25^. Meanwhile, Fig. 4b plots the variation of the tensile stress over time upon cyclic loading-unloading tests, and the results confirmed that the hybrids could withstand high stretching and almost recover the original strength upon four cyclic loading-unloadings. These results revealed that the deformation of WSCA/PAM composites was temporary and it could be recovered to its original shape and strength, an indication of self-recovery upon successive loading cycles.

## Discussion

**Figure 5:**
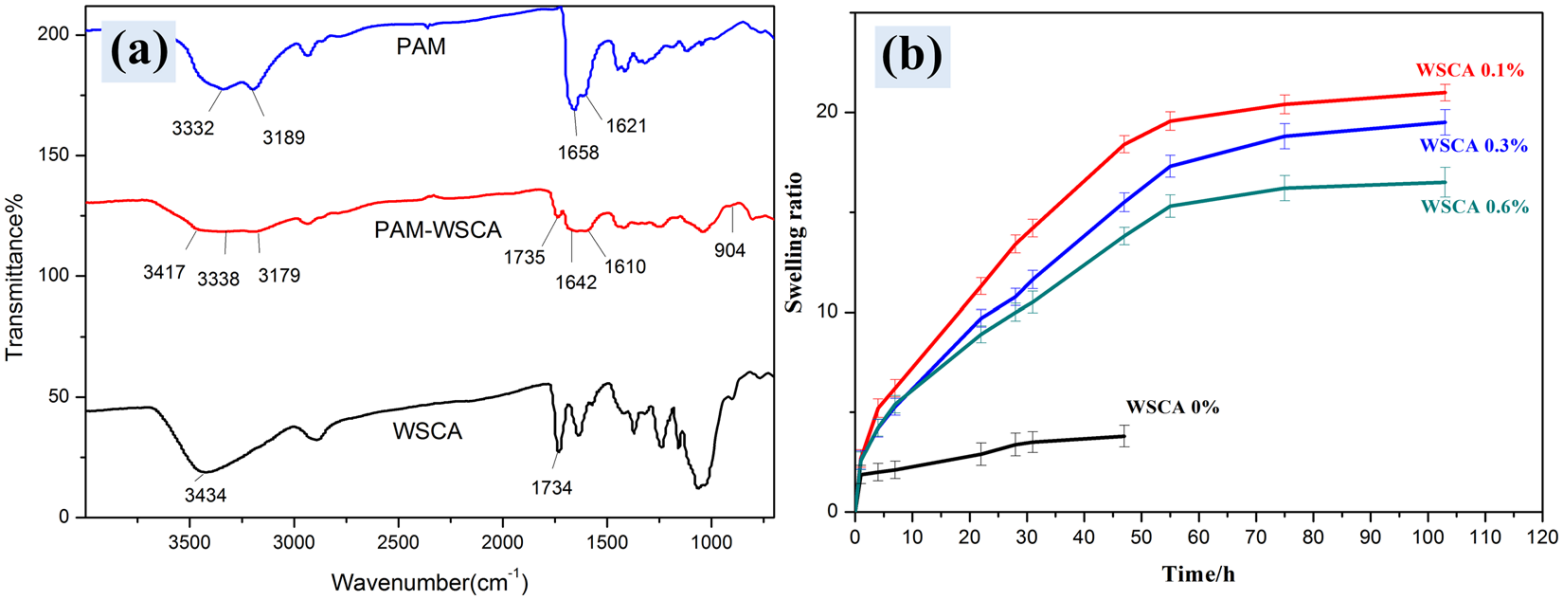
(**a**) FT-IR spectra of WSCA, WSCA-PAM gel and pure PAM gel, (**b**) Swelling behaviors of the gels with different WSCA contents.

In this work, the tough and hyperelastic gels were successfully prepared and the mechanical properties were significantly improved with the addition of WSCA. However, the reinforcement mechanism of the hybrid was still unclear. It has been reported that the continuous rigid and brittle polyelectrolyte network acted as sacrificial bonds to increase the resistance against crack propagation^26^. However, the addition of rigid and brittle cellulose to polymer matrix generally resulted in brittleness of the composites due to the poor interactions between hydrophilic cellulose and hydrophobic matrix^27,28^. In order to examine the reinforcement mechanism and interfacial interaction between WSCA and PAM matrixes, the scenario was verified as followed. Firstly, FT-IR spectrum was conducted to demonstrate the cross-linking structure in the composite hydrogels as shown in Fig. 5a. In the spectrum of WSCA/PAM composite gel, the peaks appeared at 3338 cm^−1^ and 1735 cm^−1^ were attributed to OH and C = O stretching vibration (ester bonds) of WSCA respectively^29^. Meanwhile, the characteristic peak of cellulose at 904 cm^−1^ was also detected^30^, which indicated that WSCA was actually presented in the WSCA/PAM gel samples and the composite hydrogels were successfully prepared. Furthermore, the formation of hydrogen bonds between WSCA and PAM chains was identified by the shift of the absorption peaks, in which the N-H characteristic peaks at 3332 cm^−1^ and 3189 cm^−1^ from pure PAM gel shifted to 3417 and 3179 cm^−1^ in WSCA/PAM gel sample, and while the strong absorption at 1658 cm^−1^ corresponding to the C = O stretching of the -CO-NH_2_ group, also moved to 1642 cm^−1^ in WSCA/PAM sample. Meanwhile, the peak at 1621 cm^−1^ corresponding to NH_2_ bending vibration in PAM gel transferred to 1610 cm^−1^ in WSCA/PAM gel. Therefore, the shift in the adsorption peak of -CO-NH_2_ could also demonstrate the formation of hydrogen bonds between WSCA and PAM^8^. Under deformation, the hydrogen bonds between WSCA and PAM chains were rearranged or forced to dissociate, which could dissipate energy and prevent crack occurring.

On the other hand, the existence of hydrogen bonds were also demonstrated by the swelling test (Fig. 5b). The pure PAM gel sample (WSCA 0%) was completely dissolved in water after 47 h. However, the composite gels still displayed a swelling equilibrium and did not dissolve in large excesses of water, which demonstrated the formation of hydrogen bonds between PAM chains and WSCA network. On the contrary, the hybrid gels could dissolved completely in 1 M urea solution after 48 h, indicating that the interactions between WSCA and PAM chains were pure hydrogen bonds without grafting or chemical reaction during the polymerization.

**Figure 6:**
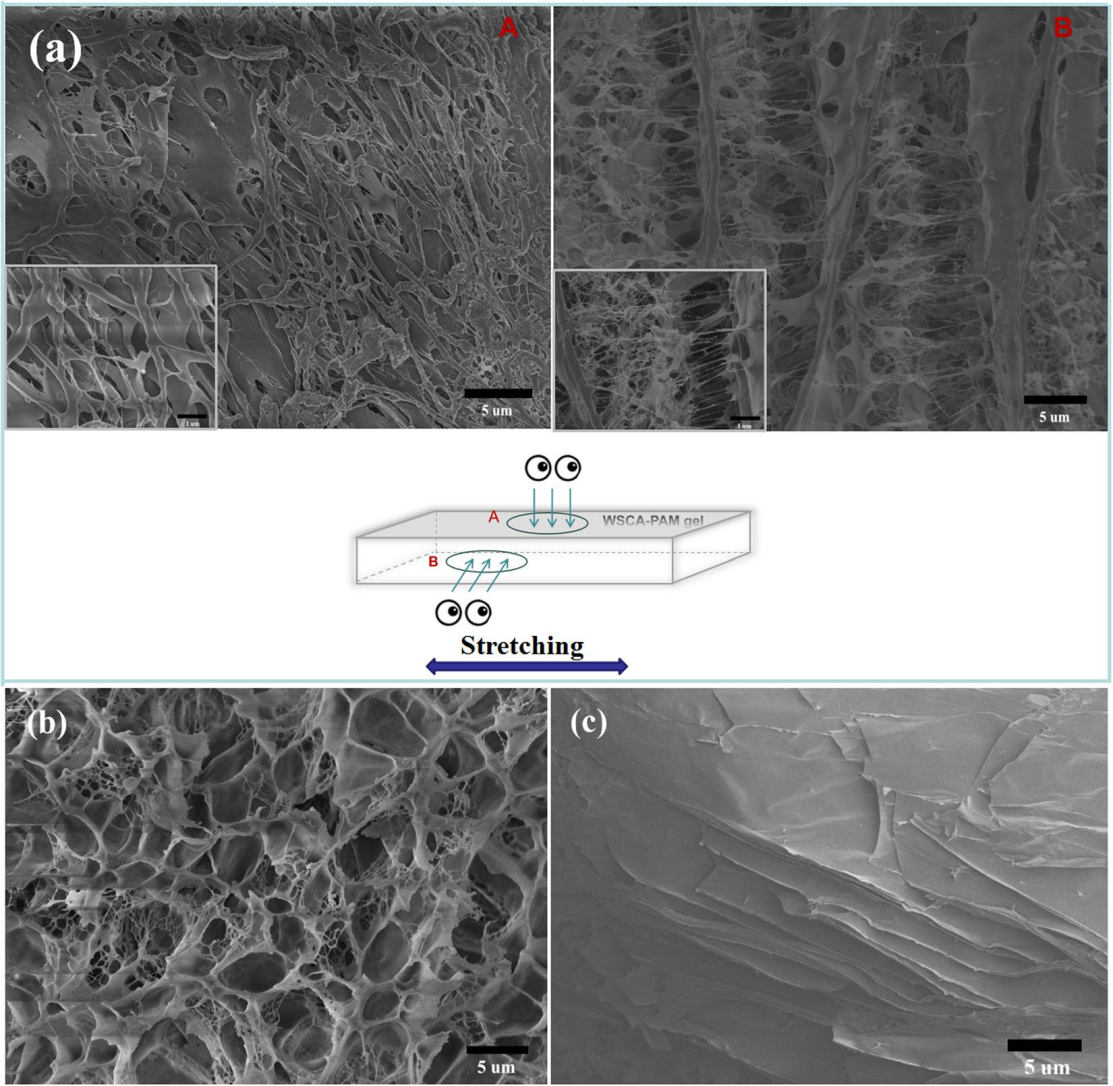
SEM images of WSCA (0.6)-PAM gels. (**a**) Surface: section A: microstructure detected to the surface of the sample; section B: cross-section to the stretching direction in the sample, (**b**) Pure PAM aerogel (by freeze-drying), (**c**) WSCA gel (0.1 g WSCA was dissolved in 10 g water and freeze-dried).

**Figure 7:**
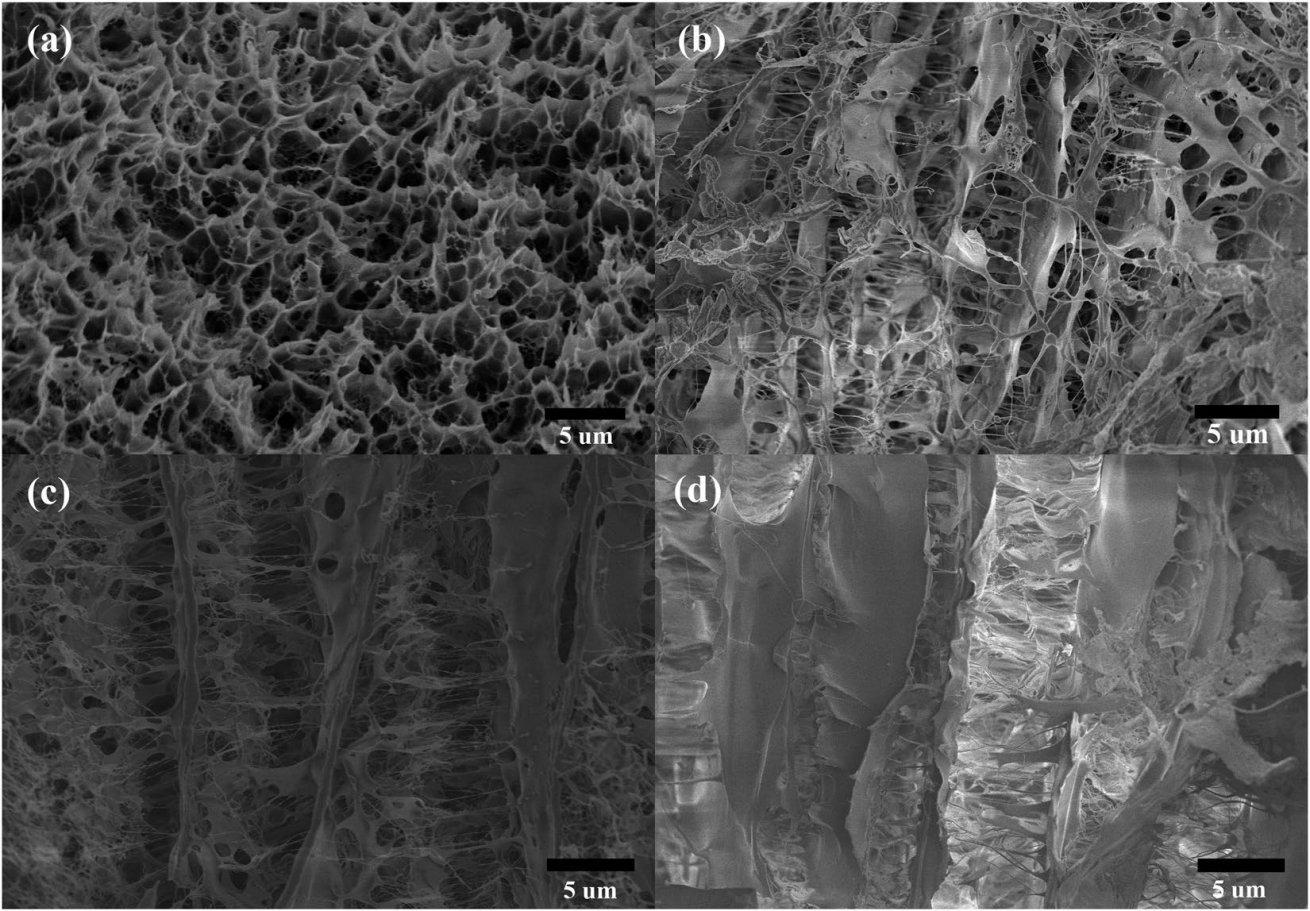
SEM images of WSCA/PAM hydrogels with different WSCA contents. (**a**) WSCA (0)-PAM gel sample, (**b**) WSCA (0.1)-PAM gel sample, (**c**) WSCA (0.6)-PAM gel sample, (**d**) WSCA (1)-PAM gel sample.

In addition, the toughening mechanisms of the hybrid hydrogels were investigated using SEM at the stretching state as shown in Fig. 6. The surface (Fig. 6A) and cross-section (Fig. 6B) morphologies of WSCA(0.6)/PAM gel were observed (Fig. 6a), and the surface properties of the pure PAM and WSCA were also comparatively characterized as shown in Fig. 6(b and c), respectively. Clearly, viewed from the above (Fig. 6a), the hybrid hydrogels exhibited cross-linked matrix networks as the WSCA and PAM were interpenetrated to each other. Moreover, the porous structure of pure PAM was observed (Fig. 6b), while the pure WSCA appeared as layered strips (Fig. 6c). Therefore, the supramolecular hybrid hydrogels was built with porous PAM and stripped WSCA, in which the WSCA acted as crossbeam while PAM was present as filler, resulting in formation of 3D network composites with numerous entanglement between the WSCA and PAM polymers (Fig. 6B). It has been widely held that the performance of polymeric materials was related to the amount of entanglement, where a higher tensile strength could be expected when the gels possessed higher amount of entanglements^31^. The presence of polymer chain coils was also demonstrated by the face-to-face multilayered structure as shown in Fig. 6B, and the layers were bridged by abundant PAM filaments. The sufficient and tight adhesion of PAM to the cellulose network matrix ensured the maximum resistance against collapse during stretching. In addition, the reinforcement of stretchability under large deformation was attributed to the multilayered and interconnected network between WSCA and PAM polymer chains in a three dimensional configuration, which could sustain splitting between WSCA and PAM matrixes along the deformation direction. The uniformly interpenetrated polymer chains between PAM and WSCA was ascribed to the homogeneous solution used for fabricating of WSCA/PAM hydrogels, in which WSCA could completely dissolve in water and it was favorable for the subsequent coordination between cellulose network and PAM matrix. Meanwhile, to further investigate the formation process of the cross-linked layered structure of WSCA/PAM composite gels, the cross-structure of WSCA/PAM hydrogels with different WSCA content are illustrated in Fig. 7. In the controlled sample (Fig. 7a), only the porous structure of PAM gel was observed. However, with the increase of WSCA concentration in the WSCA/PAM composite gels, the layered structure became more and more clearly, which demonstrated that the cross-linked layered structures between WSCA and PAM polymers were formed.

**Figure 8:**
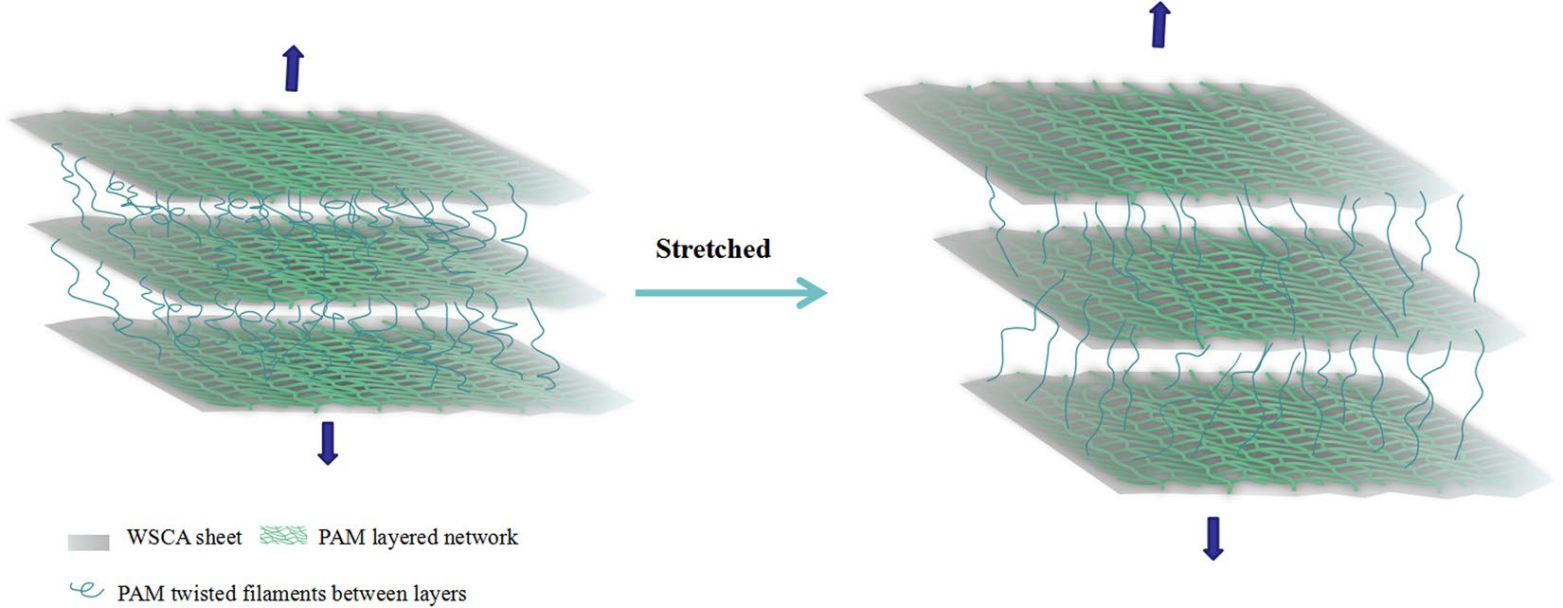
Schematic illustration of structural changes of WSCA-PAM composite gel after tensile deformation.WSCA embedded in PAM matrix or penetrated between layers, contributed to the formation of a reinforced structure, whereas physical entanglements between WSCA and PAM structure exhibited an elastic characteristics. During stretching, the initial wound fibers became unfolded or fractured and led to high flexibility.

For a better understanding of the PAM filaments embedded in WSCA sheet matrix, the schematic diagram of the structural changes of WSCA/PAM hydrogel during stretching is shown in Fig. 8. In the case of stretching, the initial filaments between layers extensively elongated or fractured, which would prevent sheet slippage and dissipate large amount of mechanical energy without compromising the structural integrity. On the other hand, this face-to-face tightly attached multilayered structure also facilitated the load dissipation in soft phase, where hydrogen bonding interactions and friction between layers allowed efficient energy dissipation under stretching. Thus, the capacity of the WSCA reinforced structure for dissipating energy under stretching was directly determined by the tremendous elongation. In such a case, the WSCA acted as the multifunctional physical cross-linker to transfer and dissipate energy during deformation and prevented crack propagation. Herein, we could state that the gratifying mechanical properties were rooted from the synergistic effect between the hydrogen bond network and physical interfacial adhesion, which, in turn, led to the formation of hybrid gels with high toughness and stretchability.

In summary, novel tough and stretchable hydrogels have been successfully synthesized by using water-soluble cellulose acetate as reinforcement. The WSCA/PAM composite hydrogels exhibited extraordinary toughness and ductility, with a tensile strength of 297 kPa and elongation at break of about 4020%. Besides, these composite gels showed extraordinary fatigue resistance against cyclic loading and the ultrahigh strains could be recovered with low residual strains upon unloading. Furthermore, the microstructure of the composite gel was also characterized, the results indicated that the three-dimensional and interpenetrating face-to-face multilayered structures were formed, which enabled to dissipate large amount of stress energy due to the slippage and disentanglement of the WSCA wound filaments.

## Experiment

### Materials

Acrylamide (AM) was purchased from Beijing Chemical Works, Ammonium persulfate (APS) was bought from West Long Chemical Co., Ltd. Cotton linter (degree of polymerization, DP = 850) was kindly supplied by Silver Hawk Fiber Corporation (Shandong province, China).Ionic liquid, 1-ethyl-3-methylimidazolium acetate (EmimAc, ≥95%), was purchased from Shanghai Cheng Jie Chemical Co., Ltd. Dichloroacetyl chloride (Cl_2_AcCl, 97%) was obtained from Alfa Aesar (A Johnson Matthey Company).All chemicals were analytical grade reagents and used as received without further purification. WSCA was synthesized as shown below.

### Synthesis of WSCA

The water-soluble cellulose acetate was prepared mainly according to our previous work^22^. Briefly, 1.00 g cellulose (6.17 mmol with respect to the repeating unit) was dissolved in 30 g EmimAc, and the slurry was kept magnetic stirring for 30 min at 80 °C, until the cellulose was dissolved completely to yield a 3 wt% solution. Then, the solution was cooled to 70 °C, Cl_2_AcCl (3.91 g, the molar ratio of reactants, i.e., dichloroacetyl chloride/anhydroglucose unit (AGU) was 3:1) was added to the cellulose solution dropwise under stirring. The reaction mixture was stirred for 24 h. Subsequently, the hot solution was poured in 200 mL ethanol, and then the modified cellulose acetate was regenerated and precipitated in the ethanol bath. After that, the modified cellulose acetate was separated by filtration and soaked in 200 mL methanol under stirring for 24 h to remove any residual ionic liquid and unreacted reagents. The product were dissolved in water and dried by freeze-drying.

Yield: 95%, DS = 0.63, determined by ^1^H NMR spectroscopy.

Elemental analysis: C 40.67%, H 6.53%, O 41.13%, Cl 0.034%.

FT-IR (cm^−1^): 3416 *ν*(OH), 1732 *ν*(C = O ester), 1244 *ν*((O) C-O, ester), 1023 *ν*(C-O-C AGU).

^1^H NMR (400 MHz, D_2_O) δ/ppm: 3.2–5.0 (AGU-protons), 3.3 (H-2), 3.5–3.8 (H-3, H-4, H-6b and H-5), 3.9 (H-6a), 4.5 (H-1), 1.8–2.1(OCOCH_3_).

^13^C NMR (400 MHz, D_2_O) δ/ppm: 173.9 (C = O), 102.6 (C-1), 78.6 (C-4), 74.9 (C-3), 74.0 (C-2), 72.9 (C-5), 62.7 (C-6 substituted), 59.9 (C-6 non-substituted), 20.3 (OCOCH_3_).

### Preparation of WSCA-PAM hydrogels

The composite gels were denoted as WSCA(x)-PAM according to the amount of WSCA (x wt% relative to H_2_O). The WSCA-PAM composite hydrogels were prepared by *in situ* free radical polymerization in aqueous media. Typically, the WSCA(0.1)-PAM composite hydrogels (WSCA: 0.1 wt% relative to water) were synthesized as follows: 10 mg WSCA was dissolved in 10 g deionized water under stirring at ambient temperature for 30 min to render an aqueous solution, then 2.5 g acrylamide monomer was added in the WSCA solution. After being stirred at ambient temperature for 0.5 h, a homogeneous mixture was obtained. Afterwards, the mixture was deoxygenated by nitrogen bubbling for 10 min and 28 mg APS was added to the mixture under stirring at 0 °C in an ice-water bath. Subsequently, the obtained mixture was transferred into molds and free radical polymerization was allowed to proceed at 40 °C for 6 h under a nitrogen atmosphere. The composition of hydrogels and their nomenclature were listed in Table 1. For all the synthesis, the amount of WSCA was varied while maintaining AM and water compositions. The prepared gels were stored at 25 °C before further treatments and tests.

**Table 1:** Composition of WSCA Gels.

Code	WSCA content (wt %)	M_WSCA_ (mg)	M_AM_ (g)	M_water_ (g)	M_APS_ (mg)
WSCA(0)-PAM	0	0	2.5	10	28
WSCA(0.1)-PAM	0.1	10	2.5	10	28
WSCA(0.3)-PAM	0.3	30	2.5	10	28
WSCA(0.6)-PAM	0.6	60	2.5	10	28
WSCA(1)-PAM	1	100	2.5	10	28

### Characterizations

Before the swelling test, the above as-prepared gel samples were subjected to further drying treatment in a vacuum oven at 65 °C for 48 h. Subsequently, the swelling ratio experiment was conducted by immersing an accurate amount of dried gel samples into an excess of water at 25 °C. The swollen gels were filtered using a 100-mesh gauze at certain time intervals, and blotted with filter paper to remove any water on the surface, then weighted. The swelling ratio of hydrogels was calculated as followed:

$${\rm{Swollen}}\,{\rm{ratio}}=\frac{{W}_{{\rm{wet}}}(t)-{W}_{{\rm{dry}}}}{{W}_{{\rm{dry}}}}\times 100 \% $$

where *W*_wet_(*t*) was the mass of the swollen hydrogel at swelling time *t* and W_dry_ was the mass of the initial dried sample. All samples were carried out three times repeatedly to ensure reproducibility.

Fourier transform infrared (FT-IR) spectra were measured on a Tensor 27 infrared spectrum instrument (Bruker, Germany) over the frequency range of 4000 to 400 cm^−1^ at 2 cm^−1^ resolution and 32 scans per sample. For SEM image, the gel samples were frozen immediately by immersing into liquid nitrogen for 5 min on the stretching state, and cut with a cold scalpel. For the cross-section observation, the samples were stick to the sample holder and mechanically fractured by being immersed into liquid nitrogen for 5 min. Then, the frozen and cut samples were dried by freeze-drying at −50 °C for 48 h. The dried samples were coated with gold palladium in a sputter coater (E-1010, Hitachi, Japan), and then were observed with a scanning electron microscope (S-3400N, Hitachi, Japan) at an acceleration voltages of 10 kV.

### Mechanical properties

Tensile mechanical measurements were performed using a Zwick Roell test machine (equipped with a 500 N load cell) at 25 °C under the test condition follows: cross-head speed = 60 mm/min, sample length between jaws = 20 mm. The dimensions of hydrogel samples were kept constant with a rectangular shape (60 mm length, 15 mm width, 5 mm height). The tensile strength (from the initial cross section of 75 mm^2^) and percentage elongation at break were recorded. To minimize dehydration, a thin layer of low viscosity of silicone oil was coated on the sample surface. Loading-unloading cycles were performed to examine the characteristic of viscoelastic processes. In addition, cyclic tests were also carried out from 0 to a maximal nominal strain (ε = 800%), and then the sample was unloaded at the 50 mm/min until zero stress was achieved. For reproducibility, at least three parallel samples were tested to ensure that no obvious difference was observed in the mechanical strength curves.
